# Expression and prognosis analysis of PAQR5 in kidney cancer

**DOI:** 10.3389/fonc.2022.955510

**Published:** 2022-08-31

**Authors:** Tao Lu, Hai-rong Xu, Wei Dong, Hui Dong

**Affiliations:** Department of Pathology, Eastern Hepatobilliary Surgery Hospital, The Second Military Medical University, Shanghai, China

**Keywords:** PAQR5, bioinformatics, kidney clear cell carcinoma, prognosis, immune infiltration

## Abstract

Progestin and adipoQ receptor 5 (PAQR5) affects the development of various malignancies and is specifically expressed in kidney. However, the role of PAQR5 in renal carcinoma remains unclear. We assessed the state of PAQR5 expression in kidney renal clear cell carcinoma (KIRC) by The Cancer Genome Atlas and Gene Expression Omnibus datasets. Moreover, immunohistochemistry was performed to observe the expressions of PAQR5 protein in tumor tissues. The relationships between PAQR5 expression and clinical characteristics were investigated by UALCAN. Gene Expression Profiling Interactive Analysis (GEPIA) and Kaplan–Meier plotter were used to analyze the effect of PAQR5 expression levels on overall survival and relapse-free survival (RFS). The re lationships between clinical characteristics and survival were also evaluated by univariate and multifactorial Cox regression. Gene Ontology term analysis, Kyoto Encyclopedia of Genes and Genomes analysis, and gene set enrichment analysis were performed on PAQR5 to explain the enrichment pathways and functions. Protein and protein interactions were explained by GeneMANIA and STRING. We also explored the relevance of PAQR5 to tumor immune cell infiltration and immunomodulatory molecules by TIMER and GEPIA. Finally, we explored the correlation of PAQR5 with the pathway proteins STATs, HIF-1α, and mTOR using the GSE40435 dataset. PAQR5 expression was low in KIRC and correlated significantly with clinical characteristics including cancer stage, tumor grade, and nodal metastasis status. Low PAQR5 expression was significantly associated with poorer survival. Cox regression analysis indicated that upregulation of PAQR5 was an independent factor for a good prognosis of KIRC. PAQR5 downregulation was associated mainly with STAT3 target upregulation, tumorigenesis, and poor differentiation. PAQR5 expression also correlated positively with B cells, neutrophils, macrophages, and dendritic cells and negatively with the infiltration of FOXP3^+^ Treg cells and the immune checkpoint molecules PD-1, CTLA4, and LAG3. Moreover, PAQR5 expression in KIRC was negatively correlated with the pathway proteins STAT1/2/3/4/5A, HIF-1α, and mTOR. PAQR5 is an excellent predictor of KIRC prognosis and may be a potential molecular therapeutic target.

## Introduction

Among the top 10 cancers in the world, renal cell carcinoma is listed as one of them, with approximately 403,000 new cases of kidney cancer worldwide in 2018 and a rapidly increasing incidence (1, 2). Renal cell carcinoma develops from renal epithelial cells, and 90% of kidney cancers are renal cell carcinomas. Approximately 80% of renal cell carcinomas are clear cell carcinomas, which are the most representative type of kidney cancer (3). The incidence of kidney cancer varies widely by gender. The incidence in men is approximately twice as high as the incidence in women, which may be due to physiological differences (4). Kidney cancer lacks early indicators, and patients are usually diagnosed at an advanced stage. Patients with advanced renal cell carcinoma with distant metastases have a 5-year survival rate of less than 10% (5). Thus, early detection and treatment is of great importance to kidney cancer patients. Finding early diagnostic indicators and new therapeutic targets is a priority in the treatment of kidney cancer.

Recently, abnormal expression of membrane progesterone receptors (mPRs) has been shown to be intimately associated with the development of carcinoma (6–8). mPRs are divided into two groups: the b5-like heme/steroid-binding protein family (MAPRs) (PGRMC1, PGRMC2, NENF, and CYB5D2) and the class II progestin and adipoQ receptor (PAQR) family (PAQR5, PAQR6, PAQR7, PAQR8, and PAQR9) (9, 10). mPRs are found in a variety of tissues and immune cells and are all seven transmembrane proteins (11, 12). mPRs are coupled to G proteins and appear to work *via* a G (i)–mediated pathway (13). Progesterone acts on multiple immune cell subtypes *via* mPRs, activates monocyte apoptosis by inducing lipopolysaccharide, and increases the expression of proinflammatory factors (11). In addition, during pregnancy, progesterone induces the production of blocking factors that suppress immune function and prevent functional antibodies from binding to antigens (14). Many studies have shown that progesterone has both pro- and anticancer effects through mPRs in breast cancer. Progesterone appears to have a crucial effect on tumor cell proliferation, metastasis, and apoptosis through these receptors, with positive or negative effects in different tumors (15–18).

**Figure 1:**
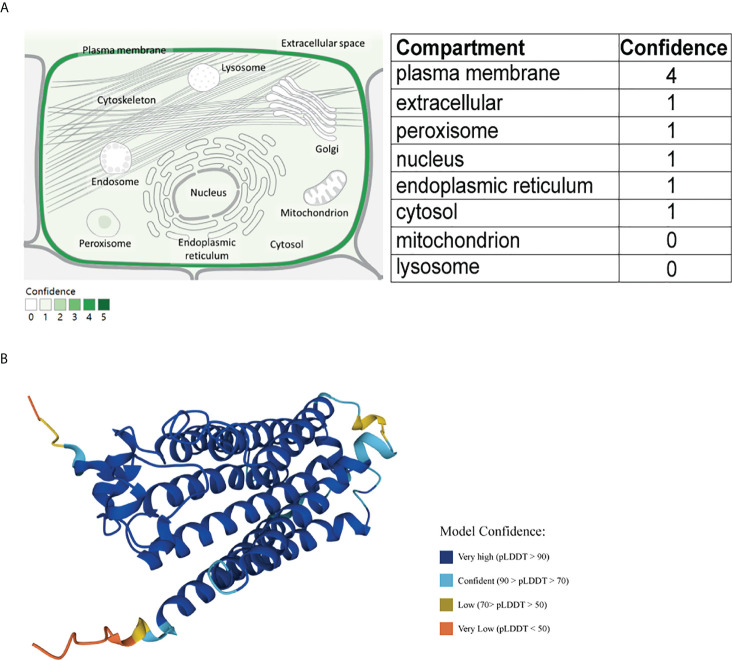
Schematic diagram of the cellular localization and protein structure of PAQR5. **(A)** PAQR5 is mainly localized on cell membranes (GeneCard). **(B)** Three-dimensional structure of PAQR5 protein (UniPort).

Among these PAQR family members, only PAQR5 is specifically highly expressed in the kidney (19, 20). PAQR5 is predicted to be located mainly in the cell membrane, and its protein structure is shown in **Figure 1**. There are no reports on PAQR family members in kidney cancer, and the significance of PAQR5 in kidney renal clear cell carcinoma (KIRC) is not yet known.

Here, we comprehensively analyzed the expression of PAQR5 in KIRC and discussed in depth its implications for clinical characteristics and prognostic value. We used the KIRC dataset from The Cancer Genome Atlas (TCGA) database for correlation analysis and the GSE40435 dataset for further confirmation. By using UALCAN, Gene Expression Profiling Interactive Analysis (GEPIA), Kaplan–Meier plotter, gene enrichment analysis, GeneMANIA, STRING, and TIMER, we systematically analyzed the significance of PAQR5 in KIRC.

## Materials and methods

### Cellular localization and protein structure of PAQR5

A cellular localization prediction map for PAQR5 was obtained from the GeneCard (https://www.genecards.org) database using the keyword PAQR5. The protein structure map was obtained in the UniProt (https://www.UniProt.org) database. GeneCard is a genecentric public database of 150 web resources providing annotations and information related to genomic, transcriptomic, proteomic, and functional investigations (21). The UniPort knowledge base contains over 60 million protein sequences and associated detailed annotations, and its resources are freely accessible to users (22, 23).

### mRNA expression analysis of PAQR5

We first performed the Wilcoxon rank sum test on PAQR5 expression data in unpaired pancarcinoma (11,093) and KIRC (611) samples that were downloaded from TCGA (https://portal.gdc.cancer.gov/) (24). Then, these samples were visualized using the ggplot2 package of R software to generate box plots. A t-test was performed on the expression data of PAQR5 in the paired samples (72 pairs) in TCGA. The Wilcoxon signed-rank test was performed on the expression in the paired samples (GSE40435, 101 pairs) (25), which were downloaded from Gene Expression Omnibus (GEO) (http://www.ncbi.nlm.nih.gov/geo) (26, 27), and a final paired plot was generated.

Receiver operating characteristic (ROC) curves were established using the pROC package to detect the value of PAQR5 for the identification of KIRC. [The area under the ROC curve ranges between 0.5 and 1. The closer the area under the curve (AUC) is to 1, the better the diagnosis; AUC values between 0.5 and 0.7 have low accuracy; AUC values between 0.7 and 0.9 have some accuracy; AUC values above 0.9 have high accuracy].

### Experimental verification of PAQR5 differential expression in KIRC tissues with immunohistochemistry

The paraffin tumor tissues and corresponding normal tissues adjacent to tumor were obtained from patients with KIRC (n = 84) at the Department of pathology, Eastern Hepatobilliary Surgery Hospital between 2016 and 2021. This study was approved by the Ethics Committee of Second Military Medical University and Eastern Hepatobilliary Surgery Hospital. Prepared 3-μm-thick tissue sections were incubated with rabbit polyclonal antibody against PAQR5 (ab236798, Abcam) for 2 h at 37°C, and the dilution ratio was 1/200. Subsequently, these were incubated with horseradish peroxidase antibody (Leica, BOND) for 1 h at room temperature and then covered by 3,3-diaminobenzidine (Leica, BOND) for 5 min. Finally, the nuclei were stained with hematoxylin (Leica, BOND) for 1 min and sealed with neutral gum. All fields of view were observed and photographed under a light microscope (Nikon Eclipse 80i, Tokyo, Japan).

### Clinicopathological variables correlation analysis

UALCAN (http://ualcan.path.uab.edu) is a one-stop database for oncology analysis, providing in-depth analysis of TCGA datasets for 31 cancers. Gene expression levels in different tumors can be queried on the basis of sample types, tumor grade, cancer stage, or other clinical features, and it is capable of calculating the association between gene expression levels and clinical characteristics (28). Here, we analyzed the correlation between PAQR5 expression levels and clinical characteristics in KIRC.

### Survival analysis

GEPIA (http://gepia.cancer-pku.cn/) provides interactive and customizable functional queries to provide users with comprehensive information through large databases such as TCGA and Genotype-Tissue Expression, thus delivering the value contained in current data resources (29). In this paper, we investigated the survival analysis of cases with low and high PAQR5 expression using GEPIA. Kaplan–Meier plotter (http://kmplot.com/analysis) is an online survival analysis tool that combines survival data from GEO, TCGA, and other databases and is designed for 21 cancer types (30). We used Kaplan–Meier plotter to validate the survival results from GEPIA.

### Univariate and multivariate cox regression analysis

Statistical analysis of TCGA survival data is carried out with survival package. To further determine the implications of PAQR5 in KIRC, we calculated the effect of PAQR5 expression levels on clinical characteristics by using univariate and multivariate Cox regression (histological grade, clinical stage, lymph node metastasis, distant metastasis, age, gender, etc.) on overall survival (OS). The clusters of PAQR5 expression are determined by median values.

### Functional enrichment and gene set enrichment analysis

We utilized the clusterProfiler package to enrich the 100 genes similar to PAQR5 collected in GEPIA and visualized them with the ggplot2 package to obtain functional annotations of biological processes (BPs), molecular functions (MFs), cellular components (CCs), and Kyoto Encyclopedia of Genes and Genomes (KEGG) pathways for PAQR5 (31).

We divided the TCGA dataset of the KIRC project into high and low groups based on the median PAQR5 expression values and performed a single-gene differential analysis of PAQR5 using the R package DESeq2 (32) and then used the clusterProfiler package for gene set enrichment analysis (GSEA) enrichment analysis and visualization by the ggplot2 package. Reference gene collection: c2.all.v7.2.symbols.gmt. Gene set database: The Molecular Signatures Database (MSigDB) (https://www.gsea-msigdb.org). The threshold for enrichment was false discovery rate < 0.25 and p. adjust < 0.05.

### Protein–protein interaction comprehensive analysis by GeneMANIA and STRING

GeneMANIA (http://www.genemania.org) is a powerful online platform to perform network construction analysis and gene function prediction for single or multiple genes of a specific species (33). We constructed a network graph using GeneMANIA for predictive analysis of PAQR5. The STRING (http://string-db.org) website can predict protein-protein interactions (PPIs) and can provide annotation information for the corresponding proteins. The new STRING database covers 5,090 organisms, 24.6 million proteins, and over 3 billion protein interactions (34, 35). Potential interactions of PAQR5 with other key proteins were identified through the construction of a PPI network analysis.

### Tumor immune infiltration analysis

TIMER (https://cistrome.shinyapps.io/timer) is focused on the investigation of immune infiltration in tumors and offers six main analysis modules that allow online exploration of the association between immune infiltration and related factors such as gene expression, clinical outcome, and cellular mutations (36). A scatter plot was generated by entering the keyword PAQR5 and looking at its expression in KIRC in relation to the infiltration of immune cells [tumor purity, B cells, CD4^+^ T cells, CD8^+^ T cells, neutrophils, macrophages, and dendritic cells (DCs)]. To investigate the mechanisms of immunosuppression, we also explored the relevance of PAQR5 expression on the surface molecules of multiple immune cell subtypes in KIRC *via* TIMER and GEPIA.

### Correlation analysis of PAQR5 with pathway proteins

To explore the relationship between PAQR5 and the JAK/STAT, VHL/HIF, and PIK3/AKT/mTOR signaling pathways, we derived expression data of PAQR5, STATs, HIF-1α, and FRAP1 in the tumor group from the GSE40435 dataset and plotted scatter plots for correlation analysis using ggplot2. The plots are statistically significant when *P* < 0.05.

## Results

### Abnormally low expressions of PAQR5 in KIRC

**Figure 2:**
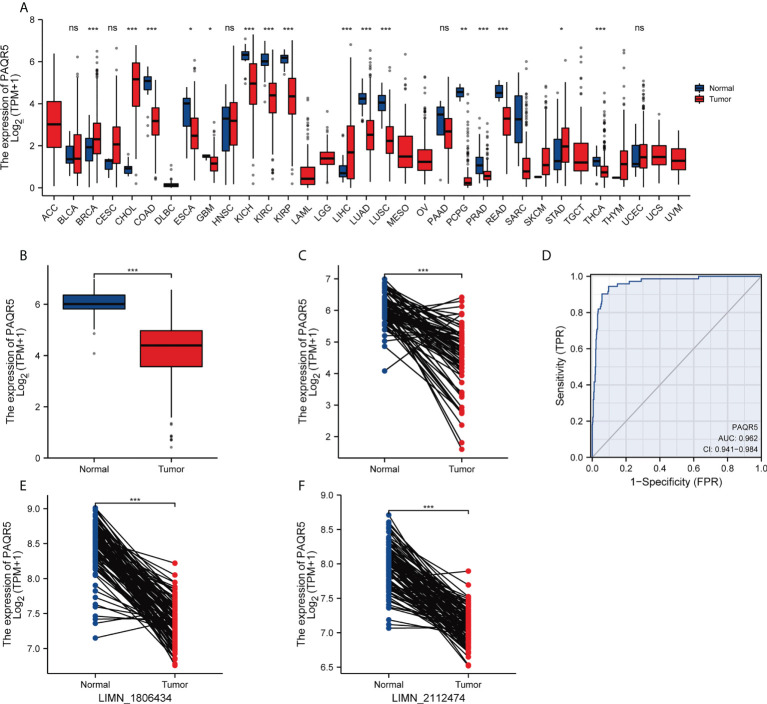
Expression levels of PAQR5 gene in different types of cancers and their paracancerous tissues. **(A)** The expression of PAQR5 was either elevated or decreased in different cancers compared with normal tissues. **(B, C)** The PAQR5 expression was significantly decreased in KIRC compared with normal tissues. **(D)** A ROC curve to test the value of PAQR5 to identify KIRC was created. **(E, F)** In the GSE40435 dataset, PAQR5 expression was significantly decreased in KIRC compared with normal tissues. Significance markers: *P < 0.05; **P < 0.01; ***P < 0.001. Ns, no statistical significance.

First, we compared PAQR5 expression in tumor and paracancerous tissues in the TCGA pancancer dataset using the Wilcoxon rank sum test. The results reveal that PAQR5 is overexpressed in liver hepatocellular carcinoma, cholangiocarcinoma, and breast invasive carcinoma. In contrast, PAQR5 was low in glioblastoma multiforme, esophageal carcinoma, colon adenocarcinoma, KIRC, kidney chromophobe, kidney renal papillary cell carcinoma, pheochromocytoma, paraganglioma, lung squamous carcinoma, lung adenocarcinoma, prostate adenocarcinoma, thyroid carcinoma, and rectal adenocarcinoma (**Figure 2A**). Then, we compared PAQR5 expression in 72 normal paracancerous tissues and 539 tumor tissues in the KIRC TCGA dataset. The results demonstrated that PAQR5 was significantly decreased in KIRC (*P* < 0.001) (**Figure 2B**). PAQR5 was also significantly downregulated in tumor tissues in 72 pairs of KIRC samples. (*P* < 0.001) (**Figure 2C**). We used ROC curves to analyze the ability of PAQR5 to distinguish between KIRC tissues and normal paracancerous tissues. As shown, PAQR5 has high accuracy in predicting tumor and normal outcomes (AUC = 0.962, CI = 0.941–0.984) (**Figure 2D**). To support this conclusion, we downloaded the GSE40435 dataset from the GEO database, which contains 101 pairs of KIRC and paraneoplastic normal tissue samples. In this dataset, PAQR5 had two reference IDs, ILMN_1806434 and ILMN_2112474, which were both expressed at substantially higher levels in normal tissues than in KIRC tissues (*P* < 0.001) (**Figures 2E, F**).

**Figure 3:**
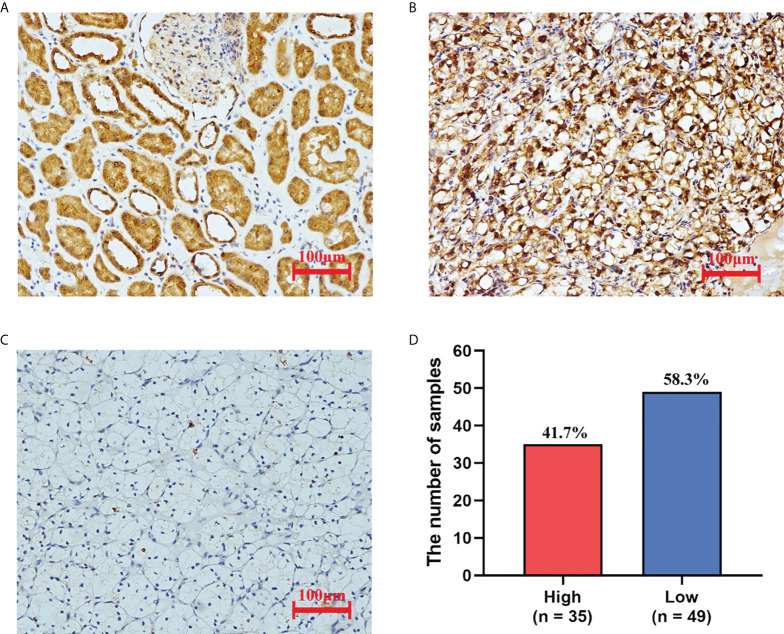
The expression of PAQR5 in KIRC (IHC). **(A)** Normal tissue adjacent to the tumor. **(B)** Tumor tissues with high PAQR5 expression. **(C)** Tumor tissues with low PAQR5 expression. **(D)** The number of samples with high and low expression.

We examined the expression of PAQR5 protein in tumor and normal tissues adjacent to the tumor by immunohistochemistry (IHC). We found that all normal renal tubular tissues (n = 84) expressed PAQR5 protein. More than half of the tumor samples (n = 49, 58.3%) did not express PAQR5 protein or just low expression compared with those highly expressed (n = 35, 41.7%) (**Figure 3**).

### Association of PAQR5 expression with clinicopathological variables

**Figure 4:**
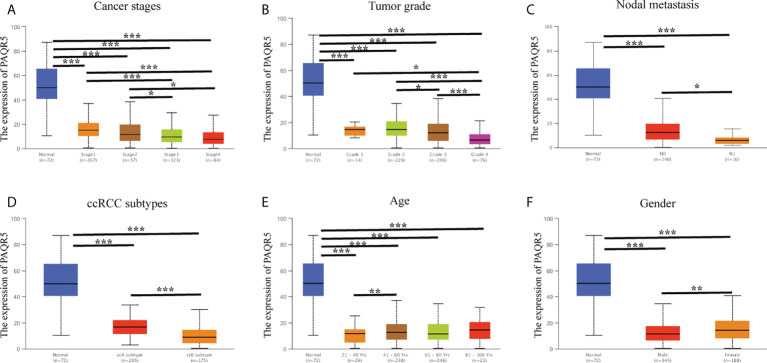
Correlation analysis of PAQR5 expression and clinical characteristics in KIRC (UALCAN). **(A, B)** PAQR5 expression was significantly and negatively correlated with cancer stages and tumor grade. **(C)** Low expression of PAQR5 was significantly correlated with nodal metastasis. **(D)** PAQR5 expression was significantly lower in the ccB subtype than in the ccA subtype. **(E)** There was no trend in PAQR5 expression with increasing age. **(F)** PAQR5 expression is higher in female than male in patients with KIRC. Significance markers: *P < 0.05; **P < 0.01; ***P < 0.001.

Here, we investigated the correlation of PAQR5 expression with cancer stage, tumor grade, nodal metastasis status, subtype, age, and gender using the UALCAN database on a KIRC dataset derived from TCGA (**Figure 4**). PAQR5 expression showed a significant decreasing trend in cancer stages, with the lowest expression in Stage 4 compared with that in Stage 1 (*P* < 0.001) (**Figure 4A**). A similar tendency was observed in the tumor grade, with the lowest PAQR5 expression value in Grade 4 compared with that in Grade 1 (*P* < 0.05) (**Figure 4B**). Tissues with lymph node metastases showed lower expression of PAQR5 than those without metastases. (*P* < 0.05) (**Figure 4C**). In the KIRC subtypes, PAQR5 expression was significantly lower in subtype ccB with more poorly prognosis than in subtype ccA with relatively better prognosis (*P* < 0.001) (**Figure 4D**). These results indicate a strong correlation between low expression of PAQR5 in KIRC tissue and poorer prognosis. In terms of gender, PAQR5 was significantly less expressed in tumor tissues from male patients than in those from female patients (*P* < 0.01) (**Figure 4F**), consistent with a higher incidence of kidney cancer in men than in women. Differences in age were seen only between the 20–40 and 40–60 groups (**Figure 4E**).

### High expression of PAQR5 predicts good prognosis

**Figure 5:**
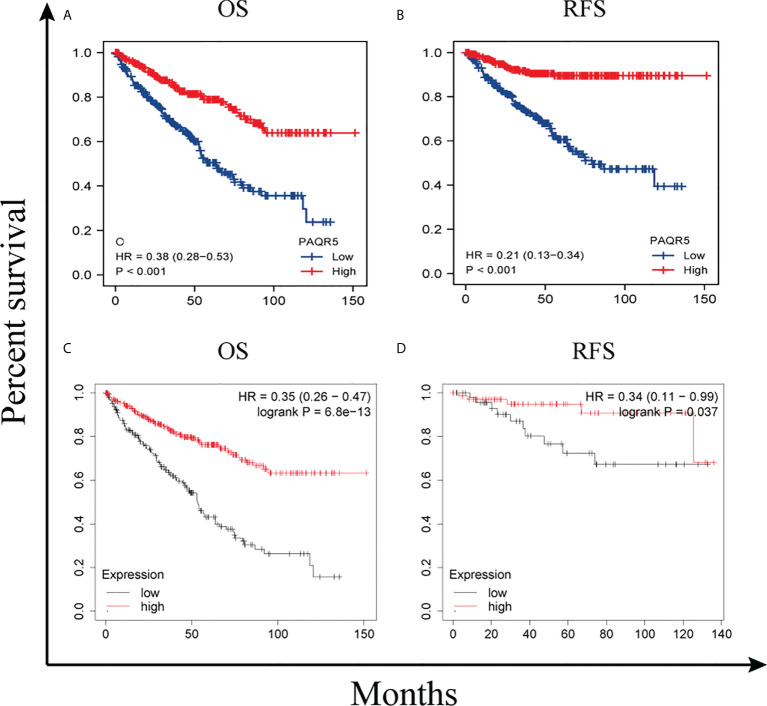
Survival curves comparing the high and low expression of PAQR5 in KIRC. **(A, B)** Survival curves of OS and RFS between PAQR5-high and PAQR5-low patients with KIRC (GEPIA). **(C, D)** Survival curves of OS and RFS between PAQR5 high and low patients with KIRC (Kaplan–Meier plotter). *P* < 0.05 was statistically significant.

Using the GEPIA online tool, we divided PAQR5 expression into high and low based on the median and analyzed its significance for KIRC survival. The results showed that the high PAQR5 expression group had better OS and relapse-free survival (RFS) (**Figures 5A, B**) (*P* < 0.001, *P* < 0.001). The same conclusion was obtained in the Kaplan–Meier plotter. Patients with low PAQR5 expression had a poorer prognosis (**Figures 5C, D**) (*P* < 0.001, *P* < 0.05). These results strongly suggest that PAQR5 is a good predictor of survival in KIRC.

To further search for factors associated with survival, we performed univariate and multifactorial Cox regression analyses for TNM stage, pathological stage, histological grade, gender, age, and PAQR5 expression. The results showed that both distant metastases and low PAQR5 expression were independent correlates of poor OS (HR, 2.648; CI, 1.568–4.474; *P* < 0.001; HR, 0.441; CI, 0.273–0.712; *P* < 0.001), DSS (disease-specific survival) (HR, 3.599; CI, 1.997–6.488; *P* < 0.001; HR, 0.221; CI, 0.106–0.461; *P* < 0.001), and PFI (progression-free interval) (HR, 4.446; CI, 2.603–7.592; *P* < 0.001; HR, 0.332; CI, 0.192–0.572; *P* < 0.001) in patients with KIRC (**Tables 1**, **S1**, **S2**).

**Table 1:** Univariate and multivariate regression (overall survival) of prognostic in patients with KIRC.

Characteristics	Total (N)	Univariate analysis		Multivariate analysis	
		Hazard ratio (95% CI)	P-value	Hazard ratio (95% CI)	P-value
T stage (T3 & T4 vs. T1 & T2)	539	3.228 (2.382–4.374)	**<0.001**	1.444 (0.632–3.297)	0.383
N stage (N1 vs. N0)	257	3.453 (1.832–6.508)	**<0.001**	1.412 (0.702–2.838)	0.333
M stage (M1 vs. M0)	506	4.389 (3.212–5.999)	**<0.001**	2.648 (1.568–4.474)	**<0.001**
Gender (Male vs. Female)	539	0.930 (0.682–1.268)	0.648		
Age (>60 vs. ≤60)	539	1.765 (1.298–2.398)	**<0.001**	1.635 (1.067–2.505)	**0.024**
Pathologic stage (Stage III & Stage IV vs. Stage I & Stage II)	536	3.946 (2.872–5.423)	**<0.001**	1.193 (0.472–3.015)	0.709
Histologic grade (G3 & G4 vs. G1 & G2)	531	2.702 (1.918–3.807)	**<0.001**	1.575 (0.947–2.622)	0.080
PAQR5 (High vs. Low)	539	0.371 (0.268–0.514)	**<0.001**	0.441 (0.273–0.712)	**<0.001**

Statistically significant at P < 0.05; HR = 1, no effect; HR > 1, increase in hazard; HR < 1, reduction in the hazard; statistically significant values are bold.

### Functional enrichment and protein–protein interaction network analysis

**Figure 6:**
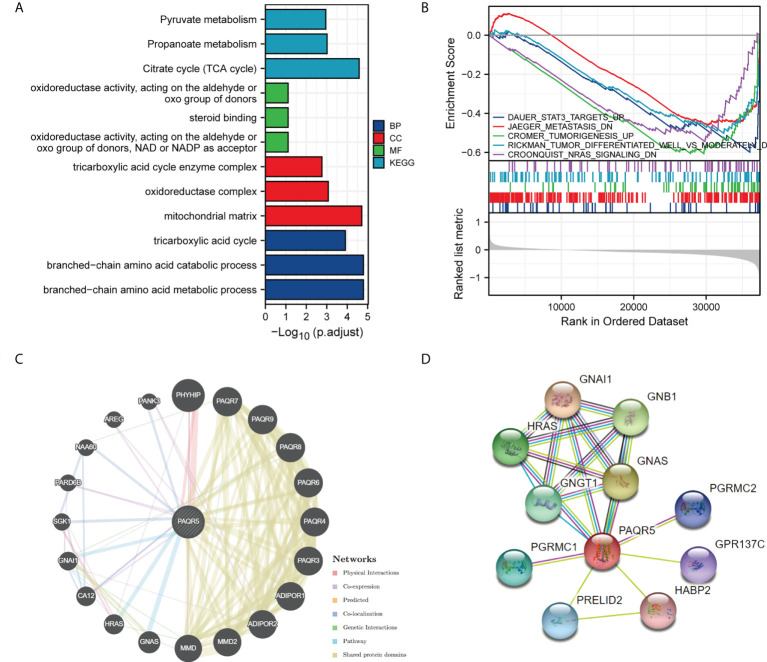
Enrichment analysis of PAQR5 in KIRC and its interacting proteins. **(A)** Enrichment analysis of GO and KEGG for similar genes to PAQR5 with bar graph. **(B)** Enrichment plot from the gene set enrichment analysis (GSEA). PAQR5 is associated with STAT3, tumor formation, metastasis, differentiation, and NRAS signaling pathway. **(C, D)** Proteins that interact with PAQR5 and their annotation information (GeneMANIA and String).

To predict the functional information of PAQR5, we collected the top 100 genes similar to PAQR5 using the similar gene functions of GEPIA. Through enrichment analysis, we obtained information on the BPs, MFs, and cellular composition involved in genes similar to PAQR5, including tricarboxylic acid cycle, branched-chain amino acid metabolic process, mitochondrial matrix, steroid binding, an oxidoreductase activity. In addition, PAQR5 may be implicated in the regulation of the citrate cycle (TCA cycle), propanoate metabolism, pyruvate metabolism, and other signaling pathways (**Figure 6A**; **Table 2**). Through GSEA enrichment analysis, we also observed that low PAQR5 gene expression was associated with upregulation of STAT3 targeting, downregulation of tumor metastasis, upregulation of tumor formation, poorer tumor differentiation, and downregulation of the NRAS signaling pathway (**Figure 6B**; **Table S3**). All these results imply that PAQR5 has important functions in the development of KIRC.

**Table 2:** The GO and KEGG function enrichment analysis of PAQR5 in KIRC.

Ontology	ID	Description	Gene ratio	p-value	q-value	Count
BP	GO:0009081	Branched-chain amino acid metabolic process	6.17%	2.61e–8	1.50e–5	5
BP	GO:0009083	Branched-chain amino acid catabolic process	6.17%	2.61e–8	1.50e–5	5
BP	GO:0006099	Tricarboxylic acid cycle	6.17%	3.42e–7	1.14e–4	5
CC	GO:0005759	Mitochondrial matrix	15.29%	1.03e–7	1.65e–5	13
CC	GO:1990204	Oxidoreductase complex	7.06%	8.91e–6	7.13e–4	6
CC	GO:0045239	Tricarboxylic acid cycle enzyme complex	3.53%	2.72e–5	0.001	3
MF	GO:0016620	Oxidoreductase activity, acting on the aldehyde or oxo group of donors, NAD or NADP as acceptor	3.75%	5.25e–4	0.068	3
MF	GO:0005496	Steroid binding	5.00%	9.02e–4	0.068	4
MF	GO:0016903	Oxidoreductase activity, acting on the aldehyde or oxo group of donors	3.75%	9.64e–4	0.068	3
KEGG	hsa00020	Citrate cycle (TCA cycle)	12.82%	2.63e–7	2.43e–5	5
KEGG	hsa00640	Propanoate metabolism	10.26%	1.94e–5	8.99e–4	4
KEGG	hsa00620	Pyruvate metabolism	10.26%	3.38e–5	0.001	4

Statistically significant at P < 0.05.

PPIs are necessary for the molecular mechanisms of tumor development and metabolic processes. GeneMANIA analysis showed that PAQR5 has pathway relationships with GNA1, HRAS, and GNAS and is coexpressed with PHYHIP, AREG, PARD6B, and CA12. PAQR5 shares protein structural domains with members of the PAQR family (**Figure 6C**). AREG proteins can inhibit the growth of certain aggressive cancer cell lines and promote the growth of normal epithelial cells by interacting with EGF/TGF-α receptors (37). STRING analysis gave us the top ten proteins and their annotations and scores for the following genes: GNA1, GNAS, GNB1, HRAS, GNGT1, PGRMC1, PRELID2, PGRMC2, GPR137C, and HABP2 (**Figure 6D**, **Table S4**). where mutations in HRAS are associated with a variety of cancers (38).

### Correlation analysis between PAQR5 expression and infiltrating immune cells

**Figure 7:**
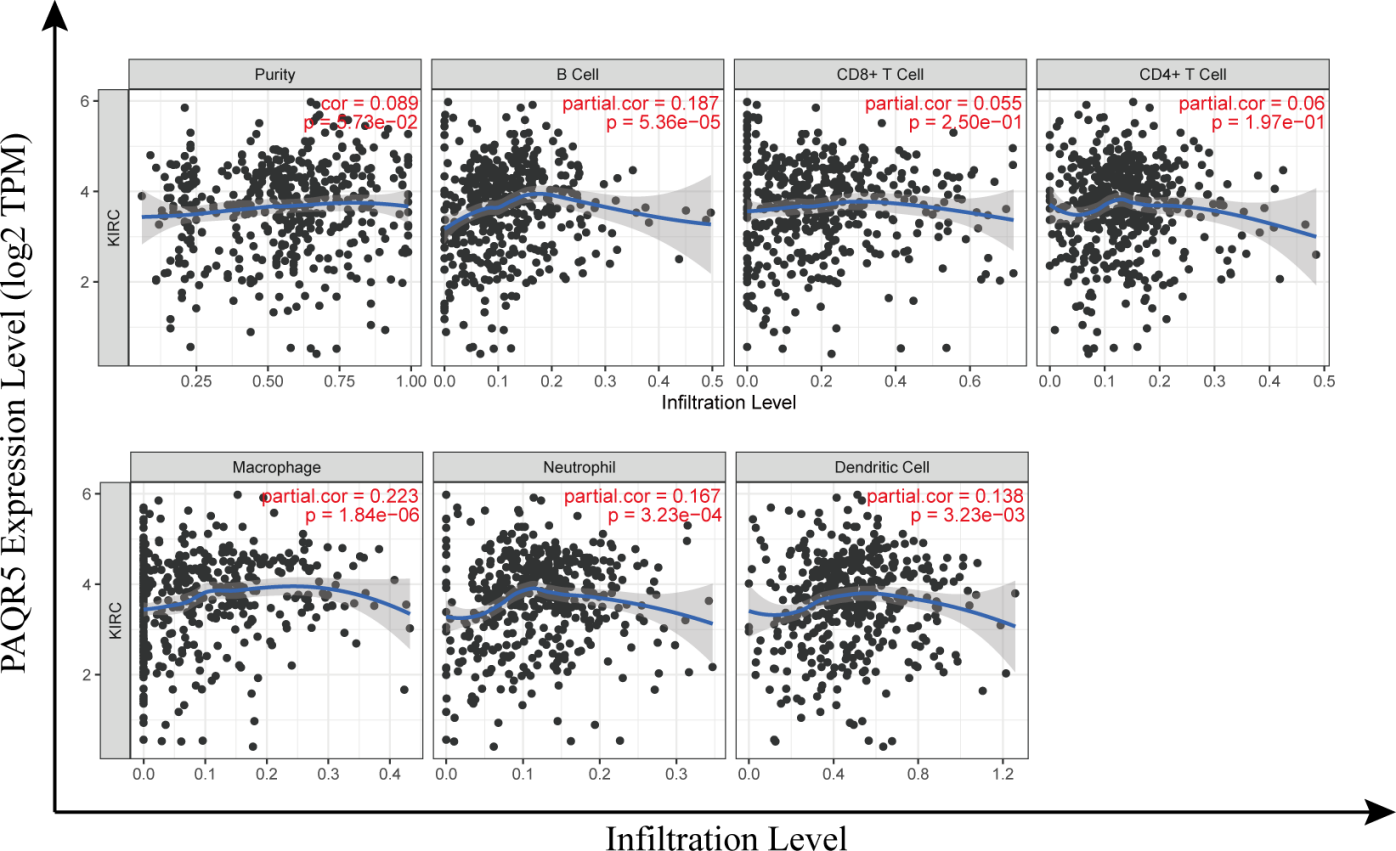
Correlation of PAQR5 expression with tumor-infiltrating immune cells in KIRC.

Kidney cancer is considered an immunogenic tumor, and there is frequently an induction of immunosuppressive cell infiltration into the tumor, leading to immune dysfunction (39). The degree of immune cells infiltration in tumor tissue is intimately related to patient prognosis and survival. We examined the correlation between PAQR5 expression and the degree of infiltration of six immune cell subtypes (CD8^+^ T cells, CD4^+^ T cells, B cells, DCs, macrophages, and neutrophils) by TIMER and its association with tumor purity. Analysis showed a statistically significant positive association with B cells (cor = 0.187, *P* = 5.36e−5), macrophages (cor = 0.223, *P* = 1.84e−6), neutrophils (cor = 0.167, *P* = 3.23e−4), and DCs (cor = 0.138, *P* = 3.23e−3) for PAQR5 expression in KIRC. No significant correlation was found with CD8^+^/CD4^+^ T cells or tumor purity (**Figure 7**), implying that antigen-presenting cells (APCs) in KIRC with low PAQR5 expression may be less expressed and unable to respond to the antitumor immune response, resulting in immune dysfunction. Numerous of studies have also shown that T-cell exhaustion is often seen in kidney cancer and is closely related to the expression of immunomodulatory molecules (40, 41). For this purpose, we deliberately explored the correlation of PAQR5 expression with Treg cells and immune checkpoint molecules. As shown in **Table 3**, low expression of PAQR5 in KIRC was obviously associated with upregulation of FOXP3^+^ Treg cells (cor = −0.247, *P* < 0.001), the immune checkpoint molecules CTLA4 (cor = −0.101, *P* < 0.05), PD-1 (cor = −0.172, *P* < 0.001), and LAG3 (cor = −0.221, *P* < 0.001), suggesting that these molecules have a leading role in immune regulation in KIRC with low PAQR5 expression and that corresponding immune checkpoint inhibitor therapy may be an effective therapeutic measure. We additionally explored the relationship between PAQR5 and the infiltration of other immune cells, comprising Tfh, Th1, Th2, Th9, Th17, Th22, TAMs, M1/M2 macrophages, and monocytes in KIRC (**Table 3**).

**Table 3:** Correlation analysis between PAQR5 and markers of immune cells in KIRC by TIMER and GEPIA.

Cell type	Gene Marker	None		Purity		Tumor		Normal	
		cor	*P*	cor	*P*	R	*P*	R	*P*
Tfh	BCL6	0.059	0.171	0.056	0.231	0.210	***	0.180	0.120
	ICOS	−0.02	0.639	0.014	0.767	0.013	0.760	−0.260	*
	CXCR5	−0.265	***	−0.23	***	−0.540	***	−0.360	**
Th1	T-bet (TBX21)	−0.039	0.371	−0.024	0.611	−0.005	0.910	−0.310	**
	STAT4	−0.089	*	−0.066	0.160	−0.043	0.330	−0.310	**
	IL12RB2	0.123	**	0.132	**	0.150	***	−0.069	0.560
	WSX1 (IL27RA)	−0.196	***	−0.177	***	−0.120	**	−0.017	0.880
	STAT1	0.212	***	0.239	***	0.290	***	0.140	0.250
	IFN-γ (IFNG)	−0.127	**	−0.115	*	−0.110	*	−0.360	**
	TNF-α (TNF)	0.029	0.499	0.051	0.274	0.085	0.051	−0.079	0.510
Th2	GATA3	−0.133	**	−0.099	*	−0.078	0.076	0.500	***
	CCR3	0.047	0.276	0.077	0.100	0.090	*	−0.340	**
	STAT6	0.416	***	0.899	***	0.400	***	0.130	0.280
	STAT5A	0.136	**	0.204	***	0.250	***	0.019	0.870
Th9	TGFBR2	0.471	***	0.466	***	0.530	***	−0.220	0.058
	IRF4	−0.069	0.133	−0.029	0.533	0.005	0.910	−0.230	0.052
	PU.1 (SPI1)	−0.188	***	−0.160	***	−0.210	**	−0.300	*
Th17	STAT3	0.426	***	0.453	***	0.510	***	0.300	**
	IL-21R	−0.167	***	−0.131	**	−0.110	**	−0.420	***
	IL-23R	0.055	0.202	0.101	*	0.110	*	−0.130	0.270
	IL-17A	−0.069	0.122	−0.023	0.619	−0.066	0.130	−0.082	0.500
Th22	CCR10	−0.269	***	−0.238	***	−0.210	***	−0.140	0.220
	AHR	0.391	***	0.408	***	0.510	***	0.080	0.500
Treg	FOXP3	−0.247	***	−0.218	***	−0.230	***	−0.078	0.510
	CD25 (IL2RA)	−0.055	0.206	−0.055	0.243	−0.008	0.850	−0.410	***
	CCR8	−0.008	0.846	0.025	0.592	0.047	0.290	−0.26	*
T-cell exhaustion	PD-1 (PDCD1)	−0.172	***	−0.158	***	−0.150	***	−0.300	*
	CTLA4	−0.101	*	−0.063	0.174	−0.067	0.130	−0.120	0.330
	LAG3	−0.221	***	−0.197	***	−0.210	***	0.048	0.690
	TIM-3 (HAVCR2)	0.239	***	0.251	***	0.280	***	−0.380	**
M1	INOS (NOS2)	0.295	***	0.381	***	0.340	***	0.091	0.45
	IRF5	0.105	*	0.135	**	0.150	***	0.310	**
	COX2 (PTGS2)	−0.017	0.701	0.016	0.728	0.077	0.078	0.150	0.220
M2	CD16 (FCGR3A)	−0.005	0.911	0.021	0.661	0.066	0.130	−0.260	*
	ARG1	0.088	*	0.067	0.150	0.150	***	−0.260	*
	MRC1	0.309	***	0.306	***	0.400	***	−0.180	0.120
	MS4A4A	0.033	0.445	0.051	0.276	0.120	**	−0.350	**
TAM	CCL2	0.109	*	0.178	***	0.150	***	−0.021	0.860
	CD80	0.025	0.562	0.06	0.199	0.055	0.210	−0.062	0.610
	CD86	0.037	0.400	0.072	0.123	0.130	**	−0.280	*
	CCR5	0.038	0.375	0.074	0.111	0.083	0.059	−0.240	*
Monocyte	CD14	−0.177	***	−0.161	***	−0.079	0.072	−0.210	0.081
	CD16 (FCGR3B)	0.202	***	0.185	***	0.220	***	−0.075	0.530
	CD115 (CSF1R)	0.045	0.298	0.077	0.0922	0.140	**	−0.230	0.055

Significance markers: P < 0.05 (*); P < 0.01 (**); P < 0.001 (***).

### Correlation analysis of PAQR5 with pathway proteins

**Figure 8:**
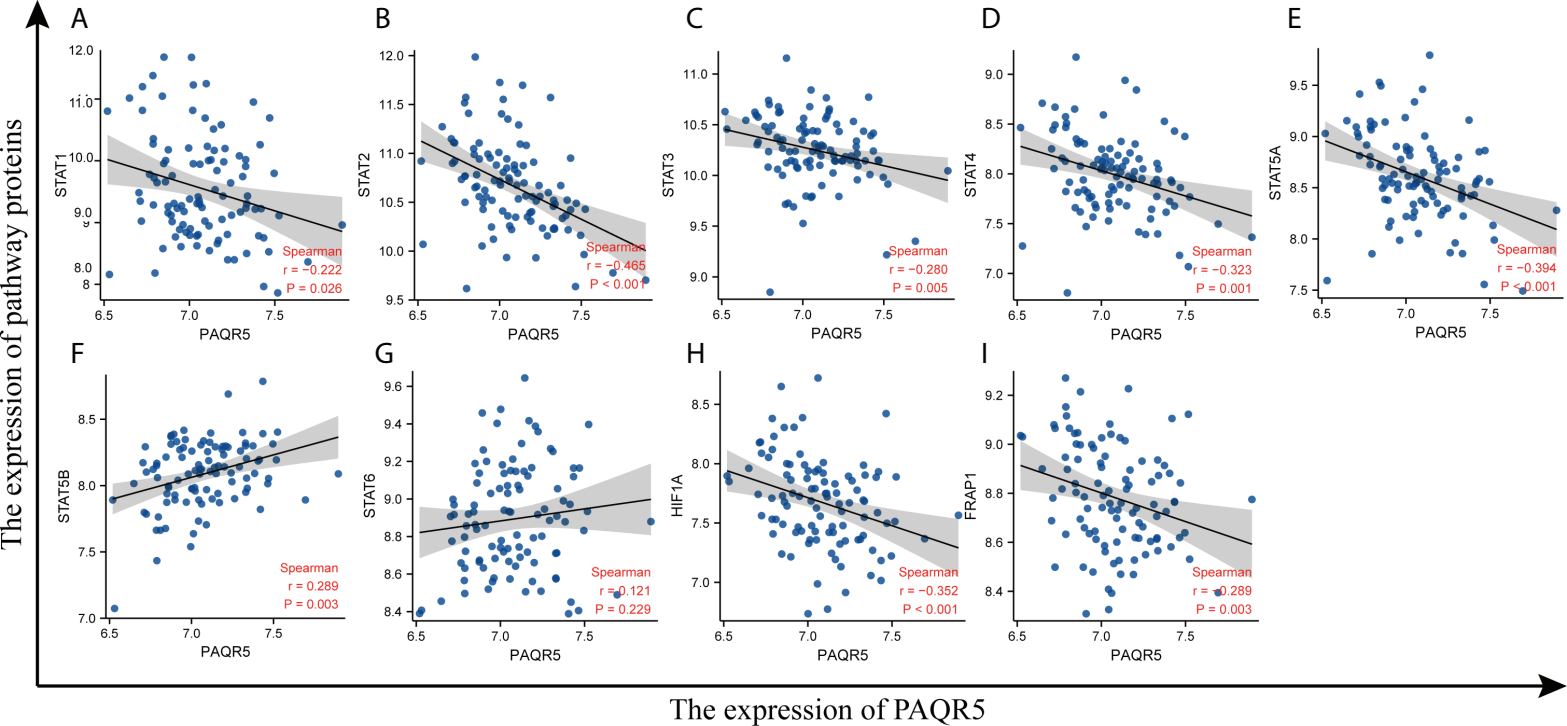
Correlation of PAQR5 expression with pathway proteins expression in KIRC. **(A–G)** Correlation of PAQR5 with STATs in KIRC. **(H)** Correlation of PAQR5 with HIF-1α in KIRC. **(I)** Correlation of PAQR5 with mTOR in KIRC.

According to reports, the common pathways activated in KIRC are PI3K/AKT/mTOR, VHL/HIF-1α, and JAK/STAT (42–44). KIRC is characterized by controlling hypoxic signaling pathways, leading to metabolic dysregulation, promoting neovascularization, and creating a microenvironment conducive to tumor growth (45). To explore which of the above pathways PAQR5 expression correlates with, we plotted a scatter plot of PAQR5 expression in KIRC against pathway proteins. First, we extracted the expression data on PAQR5, STATs, HIF1A, and FRAP1 from the GSE40435 dataset and performed Spearman correlation analysis on them separately. The scatter plot fitted curve revealed that PAQR5 was significantly negatively correlated with STAT1 (r = −0.222, *P* = 0.026), STAT2 (r = −0.465, *P* = 9.7e−07), STAT3 (r = −0.280, *P* = 0.005), STAT4 (r = −0.323, *P* = 0.001), STAT5A (r = −0.394, *P* = 4.49e−05), HIF1A (r = −0.352, *P* = 3.07e−04), and FRAP1 (r = −0.289, *P* = 0.003), positively correlated with STAT5B (r = 0.289, *P* = 0.003), and uncorrelated with STAT6 (**Figure 8**; **Table S5**), indicating that the expression of these pathway proteins, which are negatively correlated with PAQR5 expression, is increased in KIRC, implying that these signaling pathways may be in an activated state. KIRC with low PAQR5 expression may be associated with activation of the VHL/HIF-1α, JAK/STAT, and PI3K/AKT/mTOR pathways. High expression of STATs, HIF-1α, and mTOR has an important role in causing local hypoxia in tumors, forming a hypoxic barrier, tumor neovascularization, promoting tumor cell proliferation, and negatively regulating immunity and a series of other tumor-promoting events. These results also provide valuable potential therapeutic targets, and using appropriate therapeutic measures, such as improving local hypoxia in the tumor or selecting an appropriate tyrosine kinase inhibitor (TKI) could be beneficial for the prognosis of the patient.

## Discussion

To date, the differential expression of PAQR5 in KIRC and its possible prognostic values have not been discussed. This paper focuses on the expression of PAQR5 in KIRC and its potential prognostic value. We performed bioinformatic analysis of the RNA high-throughput expression matrix from the TCGA and GSE40435 datasets. The results showed that PAQR5 was transcribed significantly differently between normal kidney tissue and KIRC, which may be a good marker for KIRC. IHC results also showed that 58.3% of KIRC samples did not express PAQR5 protein. The expression of PAQR5 at the protein level was generally consistent with the mRNA level. In addition, patients with KIRC with low expression of PAQR5 have worse clinicopathological characteristics, poorer survival, and poorer prognosis. PAQR5 is strongly expressed in a diverse range of normal and cancerous tissues in a pancancer analysis and has progesterone receptor binding properties, but the signal transduction pathway activated by PAQR5 is currently unknown (9, 46, 47). It has been reported that PAQR5 expression was significantly increased in endometrial and clear cell carcinomas, and expression in normal-cycle human endometrium varies in parallel with progesterone levels (48). The expression level of PAQR5 in the kidney may also correlate with progesterone levels. To explore the potentially relevant functions of PAQR5 involved in KIRC, we conducted Gene Ontology (GO)/KEGG and GSEA analyses using the TCGA dataset. The results revealed that the downregulation of PAQR5 enriched mainly the gene set related to the upregulation of STAT3 targets, tumor formation, and poor tumor differentiation and enriched the gene set of downregulated tumor metastasis and NRAS signaling. Evidently, PAQR5 deletion or low expression may promote tumor development through the JAK/STAT signaling pathway activation. In addition, it is possible that PAQR5 may have a potentially pro-carcinogenic or anti-carcinogenic effect in different types of tumors. As seen in the PPI network, PAQR5 may influence cancer development through interactions with AERG and HRAS. PAQR5 may be a potential therapeutic target in KIRC.

Both survival and Cox regression analysis supported PAQR5 as a strong prognostic predictor for KIRC. OS, DSS, and PFI all showed poor prognosis when PAQR5 was expressed at low levels. In addition, downregulation of PAQR5 is strongly coupled with higher cancer stages, higher histological grade, metastasis, and a malignant subtype of KIRC. Low expression of PAQR5 represents a poor prognosis for patients with KIRC and is a reliable prognostic predictor.

Tumor-infiltrating immune cells are strongly associated with tumor prognosis and immunotherapy. A study has shown that KIRC has the highest immune infiltration score of the 19 cancer types, calculated according to the gene expression modality (49). Furthermore, the stage and grade of renal cell carcinoma is associated with the infiltration of helper T cells (50). Our analysis showed that in KIRC, PAQR5 expression was positively correlated with B cells, macrophages, neutrophils, and DCs. This indicates that the number of APCs in KIRC is so scarce, and it is difficult for the immune system to carry out its antitumor effects. In our study, the exhausted T-cell and FOXP3^+^ Treg-cell infiltration was higher in KIRC tissues with low PAQR5 expression. Overall, in KIRC with low PAQR5 expression, tumor-infiltrating immune cells are generally in a state of immunosuppression. The use of immunotherapy such as immune checkpoint inhibitors may be an effective measure to improve the immune microenvironment of tumors. Upregulation of PAQR5 expression may also be effective in improving KIRC immunosuppression

In addition to surgery, targeted therapeutic options such as TKIs and mTOR inhibitors have made significant breakthroughs in the treatment of KIRC. Combination immune checkpoint inhibitor therapy is a common treatment for patients with advanced kidney cancer (51). The JAK/STAT signaling pathway is a common intracellular signaling pathway involved in the regulation of cell proliferation, differentiation, apoptosis, and immune regulation. Persistent activation of the JAK/STAT signaling pathway is common in cancer, and JAK inhibitor therapy is an effective treatment for aberrant activation of this pathway (52). VHL/HIF-1α is a common signaling pathway in KIRC, and mutational inactivation of VHL leads to overexpression of HIF-1α in KIRC, inducing a tumor hypoxic microenvironment that is associated with poorer tumor-specific survival (53). Significant outcomes have been achieved in the treatment of KIRC with mTOR inhibitors, and the PI3K/AKT/mTOR signaling pathway is intimately related to the progression of KIRC and has an important role in intratumoral angiogenesis (54, 55). Our findings revealed that PAQR5 expression in KIRC was significantly correlated with STATs, HIF-1α, and mTOR. Low expression of PAQR5 in KIRC appears to be associated with a variety of adverse events. PAQR5 may have important anticancer effects in KIRC and may be a prospective critical anticancer factor. These insights provide new ideas for the treatment of KIRC.

Although these results provide further insight into the relationship between PAQR5 and KIRC, there are still limitations. Establishing a true elucidation of the relationship between PAQR5 and KIRC requires a comprehensive understanding of the entire treatment course of the patient and requires consideration of additional clinical factors that are not available in public databases. Moreover, these data come from different centers, and it is difficult to standardize the data. The bias is caused by the unequal amount of data from healthy subjects versus cancer patients. Finally, this study is based on the conclusions derived from the analysis of the TCGA dataset and the GSE40435 dataset, and the next step is necessary to explore the mechanisms of direct or indirect action of PAQR5 in KIRC. Although the treatment of renal cell carcinoma with progesterone improves prognosis (56, 57), whether the role of PAQR5 in improving the prognosis of KIRC is related to progesterone remains to be further discussed.

In our current research, we first reported that low expression of PAQR5 was related to various adverse events in KIRC, including poor survival, poor prognosis, tumor progression, and tumor immunosuppression. PAQR5 is significantly decreased in early KIRC and may be a sensitive early prognostic predictor, as well as a potential therapeutic target. The specific mechanism of action between decreased PAQR5 expression in KIRC and poor prognosis needs to be further investigated. This study provides an entry point for further investigation into the mechanisms of KIRC development, diagnosis, and treatment.

## Data availability statement

The datasets presented in this study can be found in online repositories. The names of the repository/repositories and accession number(s) can be found in the article/**Supplementary Material**.

## Ethics statement

The studies involving human participants were reviewed and approved by The Ethics Committee of Second Military Medical University and Eastern Hepatobilliary Surgery Hospital. The patients/participants provided their written informed consent to participate in this study.

## Author contributions

HD contributed to concept and design of this article. TL and H-rX participated in data collection, analysis, and manuscript writing. WD interpreted the data and proofread the article. All authors contributed to the article and approved the submitted version.

## Funding

This study was supported by grants from the National Natural Science Foundation of China (grant number: 81000969), Shanghai Municipal Health Commission (grant number: 20214Y0359), and Shanghai Science and Technology Administration Commission (grant numbers: 22Y11909100 and 22Y11908700).

## Acknowledgments

The authors would like to thank TCGA, GEO, UALCAN, Kaplan–Meier plotter, GEPIA, and TIMER for providing open access data.

## Conflict of interest

The authors declare that the research was conducted in the absence of any commercial or financial relationships that could be construed as a potential conflict of interest.

## Publisher’s note

All claims expressed in this article are solely those of the authors and do not necessarily represent those of their affiliated organizations, or those of the publisher, the editors and the reviewers. Any product that may be evaluated in this article, or claim that may be made by its manufacturer, is not guaranteed or endorsed by the publisher.
