# Supplementary material for: Expression and prognosis analysis of PAQR5 in kidney cancer
Source: Front Oncol. 2022 Aug 31;12:955510. doi: 10.3389/fonc.2022.955510 (PMC9471140; doi:10.3389/fonc.2022.955510)
Supplement: Supplementary file 1 [file Table_1.docx]

| Table S1\| Univariate and multivariate regression (Disease Specific Survival) of prognostic in patients with KIRC. | | | | | | |
| --- | --- | --- | --- | --- | --- | --- |
| Characteristics | Total(N) | Univariate analysis | |  | Multivariate analysis | |
|  |  | Hazard ratio (95% CI) | P value |  | Hazard ratio (95% CI) | P value |
| T stage (T3&T4 vs. T1&T2) | 528 | 5.542 (3.652-8.411) | **<0.001** |  | 1.191 (0.503-2.823) | 0.691 |
| N stage (N1 vs. N0) | 255 | 3.852 (1.825-8.132) | **<0.001** |  | 1.070 (0.494-2.315) | 0.864 |
| M stage (M1 vs. M0) | 495 | 9.108 (6.209-13.361) | **<0.001** |  | 3.599 (1.997-6.488) | **<0.001** |
| Gender (Male vs. Female) | 528 | 1.220 (0.807-1.845) | 0.346 |  |  |  |
| Age (>60 vs. <=60) | 528 | 1.335 (0.914-1.950) | 0.135 |  |  |  |
| Pathologic stage (Stage III&Stage IV vs. Stage I&Stage II) | 525 | 9.835 (5.925-16.325) | **<0.001** |  | 2.765 (0.928-8.240) | 0.068 |
| Histologic grade (G3&G4 vs. G1&G2) | 520 | 4.793 (2.889-7.952) | **<0.001** |  | 1.606 (0.791-3.259) | 0.190 |
| PAQR5 (High vs. Low) | 528 | 0.209 (0.130-0.337) | **<0.001** |  | 0.221 (0.106-0.461) | **<0.001** |
