# Supplementary material for: Expression and prognosis analysis of PAQR5 in kidney cancer
Source: Front Oncol. 2022 Aug 31;12:955510. doi: 10.3389/fonc.2022.955510 (PMC9471140; doi:10.3389/fonc.2022.955510)
Supplement: Supplementary file 2 [file Table_2.docx]

| Table S2\| Univariate and multivariate regression (Progress Free Interval) of prognostic in patients with KIRC. | | | | | | |
| --- | --- | --- | --- | --- | --- | --- |
| Characteristics | Total(N) | Univariate analysis | |  | Multivariate analysis | |
|  |  | Hazard ratio (95% CI) | P value |  | Hazard ratio (95% CI) | P value |
| T stage (T3&T4 vs. T1&T2) | 537 | 4.522 (3.271-6.253) | **<0.001** |  | 1.167 (0.571-2.386) | 0.672 |
| N stage (N1 vs. N0) | 256 | 3.682 (1.891-7.167) | **<0.001** |  | 0.955 (0.471-1.935) | 0.898 |
| M stage (M1 vs. M0) | 504 | 8.968 (6.464-12.442) | **<0.001** |  | 4.446 (2.603-7.592) | **<0.001** |
| Gender (Male vs. Female) | 537 | 1.515 (1.067-2.151) | **0.020** |  | 1.083 (0.691-1.699) | 0.728 |
| Age (>60 vs. <=60) | 537 | 1.275 (0.934-1.742) | 0.126 |  |  |  |
| Pathologic stage (Stage III&Stage IV vs. Stage I&Stage II) | 534 | 6.817 (4.770-9.744) | **<0.001** |  | 2.759 (1.124-6.773) | **0.027** |
| Histologic grade (G3&G4 vs. G1&G2) | 529 | 3.646 (2.503-5.310) | **<0.001** |  | 1.399 (0.824-2.377) | 0.214 |
| PAQR5 (High vs. Low) | 537 | 0.292 (0.205-0.416) | **<0.001** |  | 0.332 (0.192-0.572) | **<0.001** |
