# Supplementary material for: Expression and prognosis analysis of PAQR5 in kidney cancer
Source: Front Oncol. 2022 Aug 31;12:955510. doi: 10.3389/fonc.2022.955510 (PMC9471140; doi:10.3389/fonc.2022.955510)
Supplement: Supplementary file 3 [file Table_3.docx]

| Table S3\|Gene set enrichment analysis of PAQR5 in KIRC | | | |
| --- | --- | --- | --- |
| Gene set name | NES | p.adjust | FDR |
| DAUER_STAT3_TARGETS_UP | -1.426 | 0.042 | 0.036 |
| JAEGER_METASTASIS_DN | -1.503 | 0.042 | 0.036 |
| CROMER_TUMORIGENESIS_UP | -1.584 | 0.042 | 0.036 |
| RICKMAN_TUMOR_DIFFERENTIATED_WELL  _VS_MODERATELY_DN | -1.732 | 0.042 | 0.036 |
| CROONQUIST_NRAS_SIGNALING_DN | -1.679 | 0.042 | 0.036 |

NES: normalized enrichment score; NOM: nominal; FDR: false discovery rate.
