# Supplementary material for: Expression and prognosis analysis of PAQR5 in kidney cancer
Source: Front Oncol. 2022 Aug 31;12:955510. doi: 10.3389/fonc.2022.955510 (PMC9471140; doi:10.3389/fonc.2022.955510)
Supplement: Supplementary file 4 [file Table_4.docx]

| Table S4\|Annotation of PAQR5-interacting proteins and their co-expression scores | | |
| --- | --- | --- |
| Gene symbol | Annotation | Score |
| GNAI1 | Guanine nucleotide-binding protein G(i) subunit alpha-1 | 0.906 |
| GNAS | Guanine nucleotide-binding protein G(s) subunit alpha isoforms XLas | 0.905 |
| GNB1 | Guanine nucleotide-binding protein G(I)/G(S)/G(T) subunit beta-1 | 0.905 |
| HRAS | GTPase HRas | 0.902 |
| GNGT1 | Guanine nucleotide-binding protein G(T) subunit gamma-T1 | 0.900 |
| PGRMC1 | Membrane-associated progesterone receptor component 1 | 0.714 |
| PRELID2 | PRELI domain containing 2 | 0.694 |
| PGRMC2 | Membrane-associated progesterone receptor component 2 | 0.674 |
| GPR137C | Integral membrane protein GPR137C | 0.607 |
| HABP2 | Hyaluronan-binding protein 2 | 0.600 |
