# Supplementary material for: Expression and prognosis analysis of PAQR5 in kidney cancer
Source: Front Oncol. 2022 Aug 31;12:955510. doi: 10.3389/fonc.2022.955510 (PMC9471140; doi:10.3389/fonc.2022.955510)
Supplement: Supplementary file 5 [file Table_5.docx]

| Table S5\| The correlation of PAQR5 with pathway proteins in KIRC | | | |
| --- | --- | --- | --- |
| Group | r | R^2^ | Spearman (*P*) |
| PAQR5 vs. STAT1 | -0.222 | -0.049 | **0.026** |
| PAQR5 vs. STAT2 | -0.465 | -0.216 | **9.7e-07** |
| PAQR5 vs. STAT3 | -0.280 | -0.079 | **0.005** |
| PAQR5 vs. STAT4 | -0.323 | -0.104 | **0.001** |
| PAQR5 vs. STAT5A | -0.394 | -0.156 | **4.49e-05** |
| PAQR5 vs. STAT5B | 0.289 | 0.083 | **0.003** |
| PAQR5 vs. STAT6 | 0.121 | 0.015 | 0.229 |
| PAQR5 vs. HIF1A | -0.352 | -0.124 | **3.07e-04** |
| PAQR5 vs. FRAP1 | -0.289 | -0.084 | **0.003** |
